# The effect of high-intensity interval training on cognitive function in patients with substance use disorder: Study protocol for a two-armed randomized controlled trial

**DOI:** 10.3389/fspor.2022.954561

**Published:** 2022-12-09

**Authors:** Carolin Haberstroh, Siri Weider, Grete Flemmen, Henrik Loe, Helle Wessel Andersson, Mats Hallgren, Mats Peder Mosti

**Affiliations:** ^1^ Department of Research and Development, Clinic of Substance Use and Addiction Medicine, St Olavs University Hospital, Trondheim, Norway; ^2^ Department of Mental Health, Faculty of Medicine and Health Sciences, Norwegian University of Science and Technology, Trondheim, Norway; ^3^ Department of Psychology, Faculty of Social and Educational Sciences, Norwegian University of Science and Technology, Trondheim, Norway; ^4^ Department of Global Public Health, Karolinska Institutet, Stockholm, Sweden

**Keywords:** physical activity, exercise, high-intensity interval training (HIIT), maximal oxygen uptake, cognitive function, executive function, BRIEF-A, substance use disorder (SUD)

## Abstract

**Introduction:**

Substance use disorder (SUD) is characterized by cognitive impairment, especially executive dysfunction. Executive function is recognized as an important determinant of treatment outcome as it is associated with dropout rate, attendance to therapy and potential relapse after treatment termination. Physical activity can have beneficial effects on cognitive function, but there is still a lack of knowledge regarding potential benefits of aerobic exercise for executive function in SUD treatment. The aim of this study is to examine the effect of aerobic high-intensity interval training (HIIT) on cognitive function and the subsequent effect on treatment outcome in patients with SUD.

**Methods and analysis:**

This study is a randomized controlled trial, including men and women ≥18 years with diagnosed SUD by ICD-10. The patients will be recruited from the department for inpatient treatment at Blue Cross - Lade Addiction Treatment Center, Trondheim, Norway. Participants will be randomized 1:1 into either HIIT (3x/week) + treatment as usual (TAU), or TAU alone. Study outcomes will be assessed at baseline, after eight weeks of intervention, and at 3- and 12-months follow-up. The primary outcome is to compare the change in executive function (via altered BRIEF-A score, Behavior Rating Inventory of Executive Function-Adult) measured between the two study groups after eight weeks. Secondary outcomes include mapping of cognitive function in different subgroups (e.g. type of substance, age, fitness level), collecting self-reported information about quality of life, craving, sleep quality, etc., as well as assessing compliance to TAU and long-term treatment outcome.

**Ethics and dissemination:**

The project was approved by the Regional Ethical Committee and will be performed in accordance with this protocol and the Declaration of Helsinki. Written informed consent will be obtained from all participants prior to inclusion. This project will explore a novel approach to how exercise can be applied in SUD treatment, beyond the well-known effects on physical health. We expect to achieve new knowledge in regard to what extent HIIT can improve cognitive abilities and subsequent treatment outcome in SUD.

**Trial registration number:**

https://www.clinicaltrials.gov/NCT05324085.

## 1. Introduction

Substance use can have detrimental effects on a person's health and way of life, with negative consequences for family, friends, and participation in society. Substance use disorder (SUD) is a general term relating to harmful substance use that has evolved into abuse and addiction, and is covered by the diagnostic criteria ICD-10 and DSM-5, encompassing both illicit and licit substance use (1). According to the World Drug Report from 2021, about 275 million people worldwide used illicit drugs in 2019, whereof roughly 36 million individuals suffered from SUD (2). Almost eight times as many people (283 million) were registered having a SUD due to alcohol in 2016 (3). Patients with SUD also have high occurrence of both physical and mental comorbidities, leading to a reduced life expectancy of about 18–24 years – the most severe premature mortality gap among individuals with mental diseases (4).

A common feature in patients with SUD is impaired cognitive functioning (CF) (5–7). This particularly relates to executive functions, which for instance include decision-making, consequence analysis, impulse/self-control, and working memory (8, 9). Executive functioning is recognized as an important determinant of treatment outcome as it is associated with dropout rate, attendance to therapy sessions, and potential relapse after treatment termination (10–12). In this regard, Andersson et al. (2019) reported a relapse rate of approximately 40% three to six months after inpatient treatment in Norway (13). This constitutes a major concern and calls for new measures to overcome this challenge. Moreover, a certain degree of CF is required to benefit from verbal-based therapy forms and to learn new coping strategies, both of which are important components of SUD treatment (11). Impaired CF can not only negatively affect the treatment process itself, but also its long-term implications as for instance community integration, occupational functioning, and quality of life (14). Thus, improving CF could work as a primer for other treatment forms (e.g., psychotherapy), favoring the rehabilitation process and outcome acutely and long-term.

Physical activity (PA) can have beneficial effects on mental health and CF in healthy populations (15–17), and individuals with mental diseases (18–21), among them also patients with SUD (22, 23). A systematic review from the American College of Sports Medicine found strong evidence supporting that PA can improve CF, particularly in individuals with cognitive impairment (21). In line with this, several studies have shown that acute exercise (i.e., one single session) could improve CF in healthy individuals, an effect that was most pronounced after high-intensity aerobic exercise (24–26). Although few randomized controlled trials (RCTs) have been conducted, one RCT showed that 24 weeks of aerobic exercise improved executive function and cerebral cortical thickness in a healthy population aged 20–67 (15). Moreover, Hwang et al. (2018) reported healthy individuals with higher levels of aerobic capacity (measured as maximal oxygen uptake (V˙O_2max_)) performing better on neurocognitive tests than less fit individuals (27). Hence, these studies indicate that PA and exercise-induced fitness gains might contribute to improve CF.

Several physiological processes and biomarkers could be involved in improving CF. At the molecular level for instance, studies have suggested that brain-derived neurotrophic factor (BDNF) (28, 29), insulin-like growth factor 1 (IGF-1) and vascular endothelial growth factor (VEGF) (29, 30), blood lactate (29, 31, 32), fibronectin type III domain-containing protein 5/Irisin (33), Klotho (34), glycosylphosphatidylinositol (GPI)-specific phospholipase D1 (Gpld1) (35), and interleukin 6 (IL-6) (36, 37) are potential candidates favoring cognition, memory and learning. Insulin and glucose metabolism are other possible links, suggesting that improved insulin sensitivity and glucose handling by PA influence brain function and cognitive abilities (38, 39). Finally, fitness status (i.e., V˙O_2max_) correlated with cerebral blood flow velocity and vasomotor reactivity, and constituted a determinant of cognitive abilities (27). Those are promising findings, but the biological mechanisms underpinning the effect of exercise on CFs are still poorly understood, and warrant further exploration (40). Thus, analyzing relevant biomarkers could contribute to new knowledge to what extent they can act as physiological mediators between exercise and improved CF.

As a measure to improve somatic health in patients with SUD, aerobic high-intensity interval training (HIIT) has been implemented as mandatory part of treatment in several inpatient clinics in Norway. Aerobic HIIT has been shown to be particularly effective for improving cardiorespiratory fitness and cardiometabolic health, also in SUD inpatients (41). Additionally, higher levels of aerobic fitness have been linked to better performance on neurocognitive tests (27), and both studies with acute (24–26, 42) and chronic HIIT interventions (43) showed promising effects on CF. Since cognitive difficulties remain a critical obstacle for recovery and community integration in patients with SUD, exercise as adjunct therapy could play a vital role in restoring cognitive resources and improve treatment outcome. However, there is still a lack of RCTs examining the effects of specific training regimes on CF in this patient group, and whether such improvements can benefit other parts of the treatment. Altogether, aerobic HIIT seems to be an appropriate intervention for concurrent improvements in physical and mental health in patients with SUD.

This project will examine a novel approach to how structured exercise can be applied in SUD treatment and could add to develop new non-pharmacological treatment options. The socioeconomic burden related to substance abuse is high (44, 45), and this study may also contribute to decrease health care costs by improving the outcome of clinical treatment and integration into society. In addition, it may contribute to improved individual health and well-being among these patients. Knowledge from this trial may, therefore, provide guidance on how supervised exercise can be used as a cost-effective and holistic approach to SUD treatment encompassing both somatic and mental health, with improved quality of life as the ultimate goal. This protocol describes the study's design, aims, methodology and clinical significance.

**Aim**

The overall aim of this project is to investigate the effect of HIIT on CF and associated mechanisms, and the subsequent effect on treatment outcome in patients with SUD.

*Primary aim*

• Assess the change in executive function between an intervention group (HIIT + treatment as usual (TAU)) and a control group (TAU) after eight weeks of intervention. Executive function will be assessed using the neuropsychological test BRIEF-A (Behavior Rating Inventory of Executive Function-Adult, self-report version). This test has been established as a valid and reliable assessment of executive functions in patients with SUD (5, 6), and was therefore chosen as the primary endpoint of this study.

*Secondary aims*

• Map CF in subgroups of patients with SUD (e.g. type of substance, age, fitness level).
• Change in other neuropsychological assessments (i.e. Montreal Cognitive Assessment (MoCA), Stroop test, working memory tasks) between time points (Table 1).
• Change in scores on self-report questionnaires (e.g. substance use, mental distress, sleep quality, quality of life, etc.) between time points (Table 1).
• Assess compliance to TAU (e.g. therapeutic hours) and long-term outcome at follow-up (i.e. relapse rate).
• Analyze exercise-induced responses of neurocognitive biochemical markers.

**Table 1:** Overview of time points of measurements.

	Pre-intervention	Intervention (eight weeks)	Post-intervention	3-months follow-up	12-months follow-up
Demographic information	x				
Patient journal*	x		x		
Concomitant medications	x	x	x		
Adverse events		x			
Physical examination/test	x		x		
– Anthropometrics – Blood sample – V˙O_2max_-test					
Neuropsychological tests					
– BRIEF-A – MoCA – Stroop test – Working memory tasks	x		x	x	x
	x		x		
	x		x		
	x		x		
Questionnaires					
– Substance use, mental distress, sleep quality, quality of life	x		x	x	x
– IPAQ	x		x		

* SUD diagnosis (i.e. primary substance of use), comorbidities and therapeutic hours. SUD, Substance use disorder; V˙O_2max_, Maximal oxygen uptake; BRIEF-A, Behavior rating inventory of executive function-adult; MoCA, Montreal cognitive assessment; IPAQ, International physical activity questionnaire.

## 2. Materials and methods

### 2.1. Study design and participants

**Figure 1:**
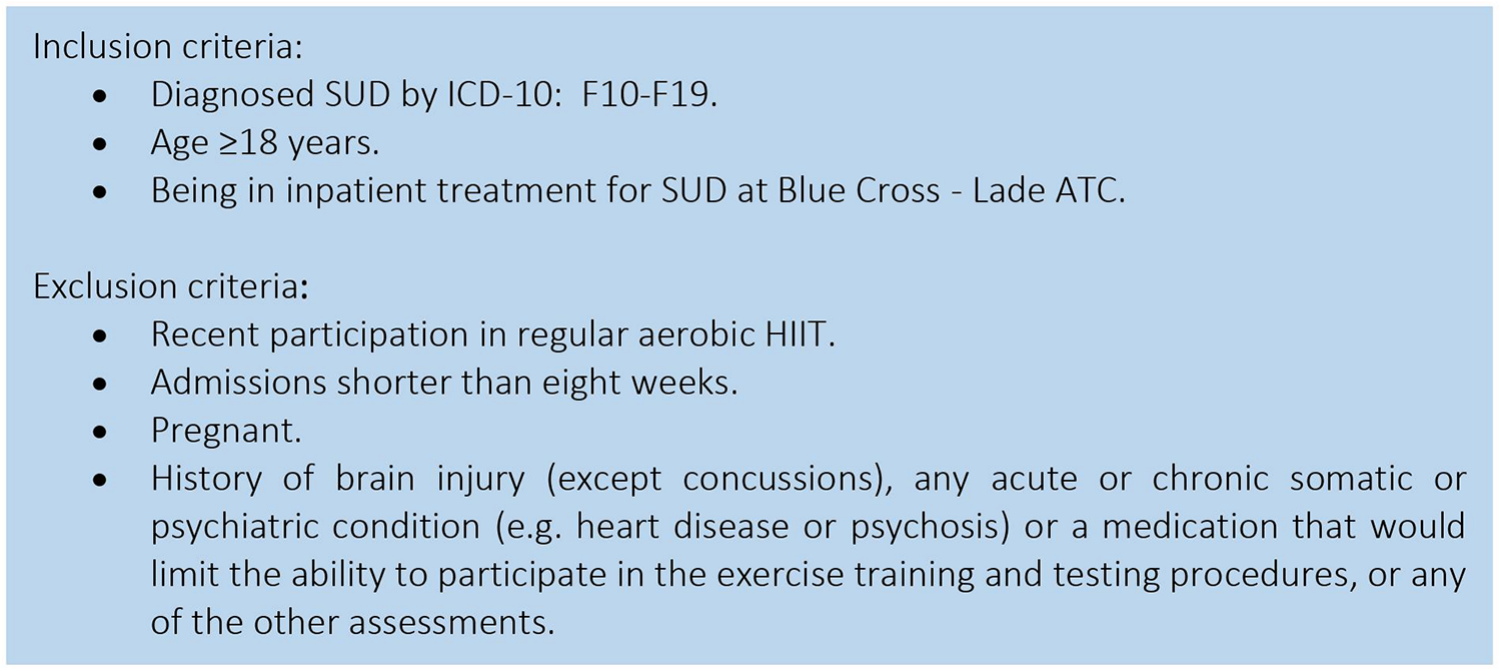
Inclusion and exclusion criteria for participants. SUD, Substance use disorder; ICD-10: F10-19 = International Classification of Diseases-10: F10-F19: Mental and behavioral disorders due to psychoactive substance use; ATC, Addiction treatment center; HIIT, High-intensity interval training.

**Figure 2:**
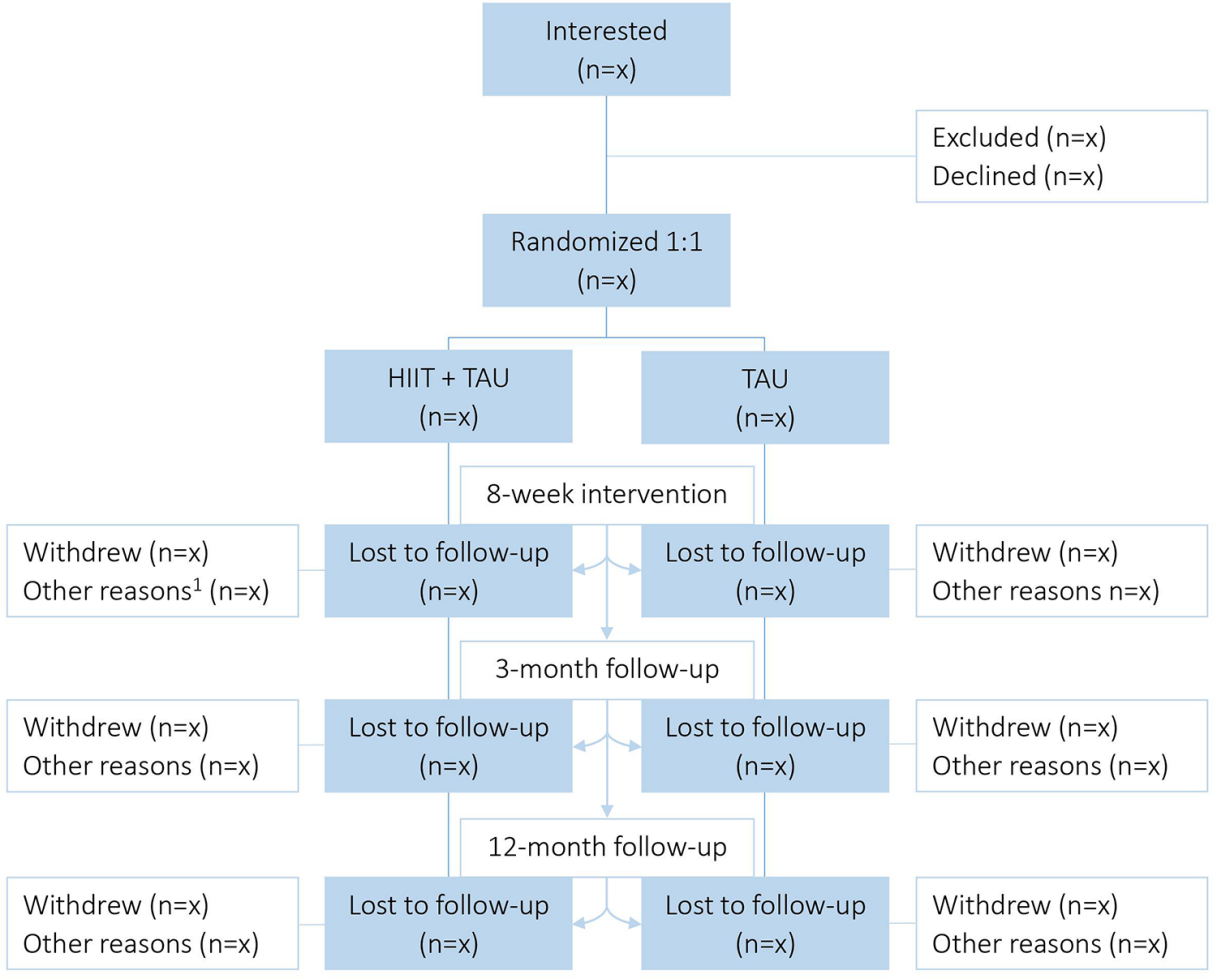
Study flow chart. HIIT, High-intensity interval training; TAU, Treatment as usual; 1 = for intervention: unable to complete/discontinuation (e.g. sick, relapse), missing data; for follow-up periods: unable to contact after ended treatment.

This study is a parallel-group RCT (superiority trial) initiated and coordinated by the Clinic of Substance Use and Addiction Medicine (CSAM), St. Olavs Hospital, Trondheim, Norway. Patients with SUD will be recruited from the department for inpatient treatment at Blue Cross - Lade Addiction Treatment Center (Lade ATC), Trondheim, Norway (inclusion and exclusion criteria see Figure 1). Eligible patients will be stratified for primary substance of use (i.e., alcohol or other) and randomized 1:1 to either aerobic HIIT + TAU, or TAU alone (Figure 2). The Clinical Research Unit of Central Norway will provide a block randomization with the software WebCRF3. Recruitment and testing of participants will be carried out by research staff affiliated CSAM, as will the supervision of the HIIT exercise sessions. Therapists and research staff at Lade ATC will inform the patients about the study after an initial detoxification phase. The research staff will then schedule to meet with the patient to provide detailed information, and schedule baseline assessments as soon as the patient has agreed to participate and has signed the written consent form. All testing and training sessions will be performed on-site at Lade ATC. Most patients are hospitalized for 8–12 weeks and will, thus, be recruited for eight weeks. Also, eight weeks of HIIT have been shown to significantly improve aerobic fitness in patients with SUD (41), and to improve executive function in healthy individuals (43).

We intend to store participant data for analyses until 2025 and will keep them anonymized for another five years. We will handle all participant data coded with an identification-number, which links the participant to a list of names. This list will be stored as an encrypted data file on a data server belonging to St. Olavs University Hospital, and only the project leader and the responsible research coordinator will have access to this list. We will use the identification-number for all assessments, tests, and analyses of the data throughout the whole project. The Regional Committee for Medical Research Ethics, Norway, has approved the protocol and procedures. We registered the study at ClinicalTrials.gov in April 2022 (Identifier: NCT05324085).

### 2.2. Assessments

The participants will be assessed at baseline, after eight weeks of intervention, and at 3- and 12-months follow-up (Table 1). We will perform most assessments on two consecutive days: neuropsychological tests at test day one, and the fitness test and questionnaires at test day two. Blood samples will be collected on a separate day.

#### 2.2.1. Neuropsychological tests

Many patients with SUD suffer from impaired cognitive functioning, especially executive dysfunction, reduced response inhibition, attention, and working memory (9, 14, 46), which can influence treatment outcome negatively (10–12). A selection of standardized and validated neuropsychological tests and work tasks will be conducted to measure the extent of these impairments at baseline and follow-up time points. The following tests were chosen due to their applicability (easy to administer, time-efficient) and established validity and reliability in this patient population (5–7, 47).

##### 2.2.1.1. BRIEF-A

The BRIEF-A is a 75-item standardized questionnaire appraising executive functions in real-life situations (48). It incorporates self-reported cognitive characteristics and collects subjective information about the ability to maintain appropriate control of emotional responses and behavior. The BRIEF-A encompasses nine sub-scales in three indexes/composite scores: *inhibit, shift, self-monitor, emotional control* (Behavioral Regulation Index, BRI); *initiate, plan/organize, working memory, organization of materials, and task monitor* (Metacognition Index, MI); BRI and MI are also combined to an overall summary score (Global Executive Composite, GEC). It additionally includes three validity scales (i.e., negativity, inconsistency, and infrequency). Conducting BRIEF-A takes 10–15 min. All items are rated with a 3-point scale, whereas a total score ≥65 points is considered as a clinically significant executive function deficiency (48, 49). The BRIEF-A has been shown to be particularly sensitive for assessing executive functions in the SUD population, and is considered more relevant for real-life situations than performance-oriented measures of executive functions (e.g., memory tasks) (5, 50).

##### 2.2.1.2. MoCA

The MoCA is a brief screening tool to detect mild cognitive impairment (51), and is used in populations with different mental and cognitive health challenges, including SUD (6, 52). It is a performance-based test that can be administered in approximately ten minutes and assesses the following CF categories: *visuospatial/executive abilities*, *naming*, *memory*, *attention*, *language*, *abstraction*, and *orientation* (51). All categories range from 3 to 5 points each, leading to a maximal score of 30 points, whereas a score of <26 points is considered as having impaired CF (51).

##### 2.2.1.3. Stroop test and working memory tasks

We will use commercially available tests developed by *Cambridge Brain Science* (https://www.cambridgebrainsciences.com/). This web-based cognitive assessment platform delivers digital cognitive tests based on validated traditional neuropsychological tasks (e.g., original Stroop test, 1935 (53)). These computer-based tests are widely used and cited in scientific literature (54, 55). As opposed to more traditional pen-and-paper tasks, they have the advantage of being more interactive and dynamic due to the task's difficulty level automatically adjusting to the participant's performance (55).

We will conduct the tests “Double Trouble” (manual Stroop task), “Digit Span” and “Token Search” to assess *response inhibition* and *selective attention*, *verbal short-term/working memory*, and *spatial working memory*, respectively. Performing these tests takes approximately five minutes each. Outcome measures are number or accuracy of correct answers and response time (56).

#### 2.2.2. Physical examination/tests

##### 2.2.2.1. Anthropometrics

Before physical testing, body weight and height will be measured with a standard scale and a stadiometer (standing without shoes), respectively. Medical staff at the clinic will perform a general health check (e.g., assess blood pressure and resting heart rate) as a standardized part of patient care. The research staff will also retrieve this information from patient journals.

##### 2.2.2.2. Exercise test

Cardiorespiratory fitness will be measured as V˙O_2max_ (or V˙O_2peak_, if criteria for V˙O_2max_ are not fulfilled, see below) by cardiopulmonary exercise testing, which is regarded as the gold standard to determine aerobic fitness level (57). Maximal oxygen uptake will be assessed on a treadmill (TX200, Gymleco, Norway) using a metabolic gas analyzer (MetaLyzer 3B, Cortex, Germany). Participants will start with a 10-min warm-up at 5% incline, followed by an individualized test protocol. Here, we will increase either incline (1%–2%) or velocity (0.5–1 km · h^−1^) every minute until exhaustion. The mean of the highest oxygen uptake values measured during a 30 s interval is defined as V˙O_2max_. At least one of the following criteria will be used to verify V˙O_2max_: plateau in oxygen uptake despite increasing workload; respiratory exchange ratio > 1.05; blood lactate concentration >7 mmol (58). Blood lactate will be measured within one minute after the test (Biosen C_line, EKF Diagnostics GmbH, Barleben, Germany). Maximal heart rate (HR_max_) will be assessed by adding two beats per minute to the highest HR attained in the test (59), and will be used as a basis to calculate the workload for the HIIT sessions. Moreover, maximal effort during the test will be evaluated by Borg Rating of Perceived Exertion (RPE) scale (60).

##### 2.2.2.3. Blood samples and immunoassays

To add biological insight, we will analyze relevant biochemical markers of neuroplasticity and cognition. Our aim is to explore possible alterations in serum concentration of relevant biomarkers of neurocognition throughout the intervention. Qualified staff at Lade ATC will draw blood samples following an overnight fast, before and after the intervention, applying well-established and validated procedures. Serum samples will be stored at −80°C until further analysis. The serum concentrations of relevant proteins (e.g., BDNF, Klotho and Gpld1) will be assessed using enzyme-linked immunosorbent assay (ELISA) kits. Additionally, we will use multianalyte profiling (Milliplex MAP) assays to assess larger panels' neurocognitive inflammatory/metabolic markers. For storage and management of serum samples, we will use the services of Biobank1 (Regional Research Biobank St. Olavs Hospital, https://biobank1.no/nb/).

#### 2.2.3. Other assessments

##### 2.2.3.1. Questionnaires

We have developed a questionnaire that acquires information concerning substance use, craving, motivation, self-esteem, sleep quality, etc. with items put together from a carefully selected battery of well-established and validated questionnaires (61–73). For instance, the 10-item short form of the Hopkins Symptoms checklist (SCL-10) has been incorporated to measure psychological distress (63), and several items of World Health organization's Quality of Life questionnaire (WHOQOL-BREF) have been added to assess quality of life (73). Additionally, we will monitor PA level by administering the International Physical Activity Questionnaire (IPAQ, short version) (74).

##### 2.2.3.2. Clinical status therapeutic hours and concomitant medication

The research staff will register demographic data and medical history. Information concerning SUD diagnosis, comorbidities, therapeutic hours and previous treatment for SUD (if applicable) will be obtained from the patient journal by authorized staff at Lade ATC.

### 2.3. Intervention

#### 2.3.1. Control group

Participants will receive standard TAU by therapists at Lade ATC with specific competence in working with patients with SUD. The content of TAU is broadly individualized, but also includes various forms of group therapy, psychotherapy, psychoeducation and PA. The PA schedule for the patients typically includes gym-based exercises (e.g., yoga, different types of circuit training, team sports) and various outdoor activities (e.g., walks, hikes, bonfire), four times per week all together.

#### 2.3.2. Exercise group

**Figure 3:**
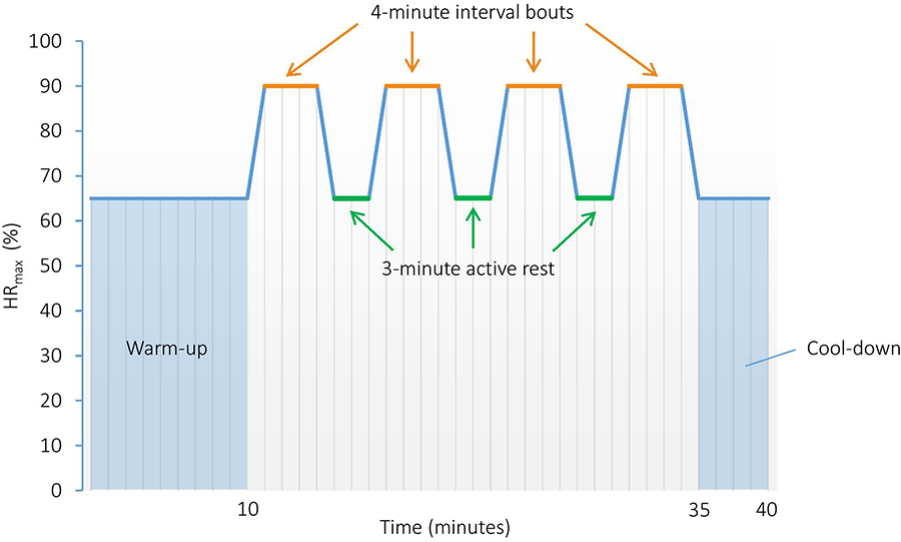
High-intensity interval training protocol performed as 4 × 4 min intervals. HR_max_, Maximal heart rate.

Participants will undergo eight weeks of supervised aerobic HIIT, three times a week. This type of training is chosen since it earlier has been shown to be effective for improving somatic health in patients with SUD (41), and to have promising effects on CF as well (43, 75). The HIIT sessions will substitute most of the PA sessions included in TAU, and will be performed as 4 × 4 min intervals, running (or walking on steep incline) on a treadmill (see Figure 3 for illustration). The session will start with a 10-min warm-up at 60%–70% of HR_max_, followed by 4 × 4-min intervals at ∼90% of HR_max_, with 3-min active rest periods at approximately 60%–70% of HR_max_ between each interval, as described previously (41). The workload is relative to the maximal exercise capacity of each patient, and will be controlled using HR monitors (Polar Vantage M multisport watch and HR sensor H10, Polar Electro, Finland) and Borg scale derived RPE (60). We will instruct the participants to follow a RPE score of 16–18 (hard–very hard) for the interval period and 11–13 (fairly light–somewhat hard) for the warm-up, active rest periods, and cool-down. Total duration of each HIIT session is approximately 40–45 min.

### 2.4. Feasibility and adherence

Intervention periods of 8–12 weeks can be considered as a normal duration for this type of training intervention (16, 41, 76). The participants will only perform three short sessions per week for eight weeks, and the training facilities are situated on-site at the clinic, with immediate access to medical assistance if needed. In addition, qualified staff will supervise all training sessions, ensuring close monitoring and facilitating adherence to training. It has already been shown good adherence to HIIT sessions in this patient population (41), confirming the feasibility of this project. Moreover, participants will exercise together two by two, providing a social aspect for training which may enhance motivation to perform and adhere to the sessions. Sport therapists at Lade ATC will facilitate the implementation of the training sessions. The exercise tests and blood sampling may cause discomfort, but these assessments are usually well tolerated when performed by experienced staff following standardized protocols. However, adverse events as fatigue, increased stress, and anxiety may occur. Patient safety is highly prioritized and in case of suspicion of adverse events or reactions, we will terminate the intervention immediately and the patient will be taken care of by medical personnel.

### 2.5. Sample size and statistical analyses

The primary comparison is altered BRIEF-A score in aerobic HIIT + TAU vs. TAU alone after eight weeks of intervention. Based on previous studies highlighting the pronounced effect of aerobic exercise, preferably using high intensity (15, 77), we anticipate a feasible effect size of  ≥ 0.6 (Cohen's *d*) on BRIEF-A score favoring the HIIT intervention. With a statistical power of 0.8 and a significance level of 0.05, it will be sufficient with 45 participants in each group. To compensate for an expected dropout rate of approximately 20%, we aim to include 55 participants in each arm of the study (*n* = 110 patients in total). This sample size is feasible to recruit within 18 months, considering the annual patient flow at Lade ATC. It will also be possible to expand the recruitment phase, and subsequent intervention period and follow-up for another six months if necessary.

We will present descriptive data as mean ± standard deviation, and medians with 25th to 75th percentiles where appropriate. To compare mean change in BRIEF-A score between the two study groups, we will apply a two-level linear mixed model (LMM), with BRIEF-A score as dependent variable, time point as level 1 and participant as level 2. Group allocation and an interaction term between group allocation and time point will be included as covariates. The LMM analyses will also produce unbiased estimate when data is missing for some time points under the assumption that the data is missing at random (78). We will perform the analysis according to the intention-to-treat principle, including all randomized study participants regardless of adherence. A per-protocol analysis of the primary outcome will also be performed, including all participants adherent to at least 70% of training sessions (i.e., 17 out of 24). We will perform similar analyses for other biological and neuropsychological parameters, as well as self-report questionnaires (secondary endpoints). In sensitivity analyses we will adjust for baseline values of cardiorespiratory fitness (27), and psychological distress (i.e., SCL-10 total score) (6). Executive dysfunction is a common feature in attention-deficit/hyperactivity disorder (ADHD), anxiety and mood disorders (79, 80), and due to high co-occurrence of those disorders among patients with SUD (81, 82), these will also be adjusted for. Statistical significance will be set to an *α*-level of *p* < 0.05, and effect sizes (i.e., Cohen's *d* (83)) will be presented together with 95% confidence intervals. We will use the software program SPSS (version 25.0 or higher) and GraphPad Prism (version 5.0 or higher) for all statistical analyses.

### 2.6. Blinding

Due to the nature of the study being an interventional trial with supervised training sessions, the study investigators and participants will not be blinded to group allocation. However, we will undertake baseline assessments prior to randomization.

### 2.7. Patient and public involvement

The project has been presented for the user committees at Lade ATC and St. Olavs University Hospital, who have commented on the study design and research questions; they have given full support for the project to be carried out. There is also one user representative in the steering group of the project, along with other representatives from CSAM and Lade ATC. To obtain valuable feedback and improve compliance to the exercise intervention and follow-up assessments, we will continue to meet and discuss with the user committees throughout the study period.

We will encourage participants to ask questions about the trial; it is of high importance to provide them with clear information, hereby communicating that personal data will be handled and stored coded, participation is voluntary, and withdrawal is possible at any point of time. Also, we will make individual test results available for each participant after testing (e.g., V˙O_2max_ test results).

## 3. Discussion

Substance use disorder is one of the most common mental disorders (84), and accounted for more than 700 substance-use-related deaths in Norway in 2020 (85). Physical activity as an adjunct to cognitive and pharmaceutical therapy in SUD treatment has been introduced to clinics about 40 years ago, with walking, games, sports, and weight training being the most reported PA programs (86, 87). However, the lack of a detailed description of interventions (e.g., frequency, volume, intensity) makes the PA programs seem unstructured and inconsistent (41, 87). Additionally, different methodological approaches, training regimes and outcome measures of trials examining PA for patients with SUD make them difficult to compare and draw clear and compelling conclusions.

The relationship between HIIT and mental health/CF is inconclusive. One RCT by Flemmen et al. (2014) looked into the effects of HIIT on somatic and mental health in patients with SUD (41). The HIIT group had significantly higher cardiorespiratory fitness than the control group after eight weeks of intervention, but the authors could not find significant between-group differences in mental health aspects such as anxiety, depression and insomnia. As stated by Flemmen and colleagues, one reason could be that the study was underpowered in order to investigate changes in mental health. Moreover, they did not include any assessments of CF. A systematic review by Ai et al. (2021) analyzed effects of HIIT on executive function and found facilitating effects, but the authors only included studies performing one acute bout of exercise in healthy participants (42). Those results are promising, but there is still limited research available when it comes to effects of aerobic high-intensity exercise on CF, especially in patients with SUD.

Data from this trial will contribute to new knowledge to what extent HIIT can have a positive impact on CF in patients with SUD specifically. If proven beneficial, HIIT can be used as a non-pharmaceutical, cost-effective and holistic approach to SUD treatment, encompassing both mental and somatic health. Knowledge from this trial can thereby contribute to reduce the economic burden on the society and health care system and improve the individual's quality of life substantially. Finally, new insight regarding exercise-induced responses of relevant hormones and cytokines involved in neurocognitive processes can also be expected, potentially broadening the mechanistic understanding of exercise-mediated alterations in brain function on the physiological level.
